# Microbe Profile: *Bdellovibrio bacteriovorus*: a specialized bacterial predator of bacteria

**DOI:** 10.1099/mic.0.001043

**Published:** 2021-04-12

**Authors:** Andrew L. Lovering, R. Elizabeth Sockett

**Affiliations:** ^1^​ Dept of Biosciences, University of Birmingham, Birmingham, UK; ^2^​ University of Nottingham, Nottingham, UK

**Keywords:** predatory bacteria, bdellovibrio, molecular microbiology

## Abstract

*
Bdellovibrio bacteriovorus* is an environmentally-ubiquitous bacterium that uses unique adaptations to kill other bacteria. The best-characterized strain, HD100, has a multistage lifestyle, with both a free-living attack phase and an intraperiplasmic growth and division phase inside the prey cell. Advances in understanding the basic biology and regulation of predation processes are paving the way for future potential therapeutic and bioremediation applications of this unusual bacterium.

## Taxonomy

Domain Bacteria, phylum Proteobacteria, class Oligoflexia, order Bdellovibrionales, family Bdellovibrionaceae, genus Bdellovibrio, species *Bdellovibrio bacteriovorus *, strain HD100. Prior to the establishment of the Oligoflexia, *Bdellovibrio* was placed in the Deltaproteobacteria; a proposal has been made to reclassify into a distinct phylum, Bdellovibrionota.

## Properties

*Bdellovibrio bacteriovorus* is a predator of other Gram-negative bacteria. The type strain HD100 was isolated from soil, and utilizes whole cell invasion, with a staged lifecycle wherein it establishes itself in the periplasmic space of the host and kills it from within [1]. Related strains can be found in diverse environments (seawater, freshwater, digestive tracts) and adopt a similar endoperiplasmic method of predation, or the seemingly less complex method of epibiotic attachment and killing via external attachment only (* B. exovorus *). HD100 can be converted into a form competent for slower laboratory axenic growth via point mutations and culturing on amino-acid rich media. Some *B. bacteriovorus* strains such as *Tiberius* alternate naturally between slower axenic growth and predation. *Bdellovibrio* cells are small (0.2–0.5 by 0.5–2.5 μm), vibroid with a monopolar, membrane-sheathed flagellum, relatively deformable during prey invasion and polymorphic in axenic growth, and they possess non-standard, mannosylated LPS.

## Genome

The complete 3782950 bp genome of HD100 is relatively large and testament to the need for *Bdellovibrio bacteriovorus* to survive in a free-living stage inbetween prey killing events and for a percentage of its population to exist replicating slowly axenically as mutants [2]. As a specialized predator, there is some expected gene loss, largely in biosynthetic pathways including those of several amino acids. A significant proportion of novel hypotheticals (approximately a third of 3584 protein coding genes) encode proteins adapted for the unique predatory lifestyle *e.g*. a lysozyme variant that assists prey cell exit [3]. Likewise, a large fraction of the proteome is predicted to be secreted, consistent with deployment of enzymes into prey.

## Phylogeny

*Bdellovibrio* have both predatory (* Myxococcus* xanthus) and non-predatory (*Desulfovibrio, Geobacter*) relatives. There is some evidence to suggest that gene transfer to *Bdellovibrio* comes largely from non-prey bacteria including Firmicutes. It is currently unclear as to the exact relationships/evolutionary path between endoperiplasmic and epibiotic strains, given that the latter can possess both similarly-sized genomes to HD100 (*e.g. B. qaytius*) or a relatively reduced genome (*e.g. B. exovorus*). Comparison between differing modes of predation, or to phylogenetically distant predators with a similar mode of killing (the Alphaproteobacterium *Micavibrio aeruginosavorus*) reveals that predator genome commonalities are mainly metabolic and that predation adaptations/mechanisms are likely to be predator-specific.

## Key features and discoveries

The staged lifecycle of predation is rich in cryptic, biologically interesting events, and begins with an attack phase in which the highly-motile *Bdellovibrio* collides with prey. An initially reversible attachment period progresses to dedicated invasion, in which the predator enters, through the prey outer membrane, sealing it neatly ‘behind’. Consumption of mainly cytoplasmic, prey macromolecules then drives growth of the now-periplasmic predator (residing in a rounded, softened, dead host-cell, termed the bdelloplast). The predator elongates as the periplasm:cytoplasm ratio increases. Dependent on prey cell size, *Bdellovibrio* cells are able to divide into a variable number of progeny, odd or even, via synchronous filamentous septation. These progeny then mature, lyse the host cell and begin the cycle anew. Recognition between predator and prey is likely multifactorial, and unlike bacteriophage, susceptible prey populations do not develop genetic resistance to killing.

An initial burst of interest in *Bdellovibrio* was hampered by the available genetic tools and alternative of more tractable bacterial systems for study. In recent years this has changed, partly through the interest in the possibility of using *Bdellovibrio* and related predators as a ‘living antibiotic’ solution to problematic bacteria in both healthcare and agriculture. One large-scale initiative to study the feasibility of this idea was funded by the US research agency DARPA (https://www.darpa.mil/program/pathogen-predators). It is now apparent that *B. bacteriovorus* is able to kill prey regardless of antimicrobial resistance gene status [4]. Allied to this, injections of *B. bacteriovorus* were demonstrated to be effective in a zebrafish model of infection, working in tandem with the immune system to reduce pathogen numbers [5]. Pathogen killing has also been demonstrated inside rats, mice and chicks.

For the purposes of economy and exclusive access to the nutrients resulting from killing the prey cell, it is important that *B. bacteriovorus* crafts a stable intraperiplasmic niche and avoids premature lysis of the host cell. A swathe of prey-cell wall-manipulating enzymes assist in these tasks, relaxing host cell peptidoglycan to soften the invaded cell and signal occupancy to kin, reducing wasteful secondary invasion events. *B. bacteriovorus* itself also has peptidoglycan and must be careful not to act against self during predation. One of several mechanisms for this is to utilize an ankyrin-based immunity protein to block action of peptidoglycan endopeptidases; the importance of this is outlined in mutants that lack this protection and self-lyse upon invasion [6]. The use of fluorescent d-amino acid reporters to illuminate cell wall modifications revealed that a ‘porthole’ is built to support passage of *B. bacteriovorus* through the prey envelope. Two further modifications of the prey peptidoglycan serve to reseal this pore and provide general reinforcement of the invaded cell [7].

Use of fluorescence microscopy has also begun to provide details on the intracellular developmental stages of the predator. *B. bacteriovorus* cells place a block on replication, when external, between kills, and it was thought that a complete round of chromosome replication was required during a singular killing event. However, a detailed study of replication timing revealed that if the host cell provided insufficient resources for this, *B. bacteriovorus* could stall before a full replication cycle occurred and then complete this during killing of a second cell [8]. Other adaptations to maximize efficient use of the host cell material include cyclic nucleotide signalling that prey is sensed, inducing predatory processes, and uptake of nucleoside monophosphates without need for breakdown into their component units.

## Open questions

- Which (surface?) features of prey determine susceptibility to *Bdellovibrio *?
- By what means is the decision to invade or not determined?
- What is the mechanistic basis for whole cell invasion, namely controlled entry through the outer membrane of prey?
- How is the remarkable variable number of progeny and resulting differential pattern of septation controlled?
- What are the key signalling events/molecules that inform on exhaustion of prey cell resources pre-exit?
